# Targeted inhibition of eIF4A suppresses B-cell receptor-induced translation and expression of MYC and MCL1 in chronic lymphocytic leukemia cells

**DOI:** 10.1007/s00018-021-03910-x

**Published:** 2021-08-16

**Authors:** Sarah Wilmore, Karly-Rai Rogers-Broadway, Joe Taylor, Elizabeth Lemm, Rachel Fell, Freda K. Stevenson, Francesco Forconi, Andrew J. Steele, Mark Coldwell, Graham Packham, Alison Yeomans

**Affiliations:** 1 Cancer Research UK Centre, Cancer Sciences, Faculty of Medicine, University of Southampton, Southampton General Hospital, Somers Building, Southampton, SO16 6YD UK; 2 School of Biological Sciences, Faculty of Environmental and Life Sciences, University of Southampton, Southampton, UK

**Keywords:** mRNA translation, eIF4A, Silvestrol, Rocaglamide, MYC

## Abstract

Signaling via the B-cell receptor (BCR) is a key driver and therapeutic target in chronic lymphocytic leukemia (CLL). BCR stimulation of CLL cells induces expression of eIF4A, an initiation factor important for translation of multiple oncoproteins, and reduces expression of PDCD4, a natural inhibitor of eIF4A, suggesting that eIF4A may be a critical nexus controlling protein expression downstream of the BCR in these cells. We, therefore, investigated the effect of eIF4A inhibitors (eIF4Ai) on BCR-induced responses. We demonstrated that eIF4Ai (silvestrol and rocaglamide A) reduced anti-IgM-induced global mRNA translation in CLL cells and also inhibited accumulation of MYC and MCL1, key drivers of proliferation and survival, respectively, without effects on upstream signaling responses (ERK1/2 and AKT phosphorylation). Analysis of normal naïve and non-switched memory B cells, likely counterparts of the two main subsets of CLL, demonstrated that basal RNA translation was higher in memory B cells, but was similarly increased and susceptible to eIF4Ai-mediated inhibition in both. We probed the fate of *MYC* mRNA in eIF4Ai-treated CLL cells and found that eIF4Ai caused a profound accumulation of *MYC* mRNA in anti-IgM treated cells. This was mediated by *MYC* mRNA stabilization and was not observed for *MCL1* mRNA. Following drug wash-out, *MYC* mRNA levels declined but without substantial MYC protein accumulation, indicating that stabilized *MYC* mRNA remained blocked from translation. In conclusion, BCR-induced regulation of eIF4A may be a critical signal-dependent nexus for therapeutic attack in CLL and other B-cell malignancies, especially those dependent on MYC and/or MCL1.

**Supplementary Information:**

The online version contains supplementary material available at 10.1007/s00018-021-03910-x.

## Introduction

RNA translation is the most energetically demanding cellular process and is tightly controlled by intra- and extra-cellular signals which predominantly act to regulate initiation of translation by the eIF4F complex [1, 2]. Efficiency of translational initiation is influenced by multiple variables, including abundance of mRNA and availability of eukaryotic initiation factors (eIFs) and ribosomal subunits, as well as primary and secondary structural features within individual mRNAs which confer mRNA-specific regulation. For example, mRNAs with highly structured 5′-UTRs require unwinding to allow efficient translation and this is mediated by the helicase, eIF4A, a core component of eIF4F [3–5].

Regulation of RNA translation is often dysregulated in cancer and targeted inhibition of specific oncogenic mRNA translation is a novel approach for anti-cancer treatment [6]. Many oncogenic proteins, such as MYC, a master regulator of cell growth and metabolism, and MCL1, a BCL2-family-related survival protein, can be dependent on the helicase activity of eIF4A for their expression [7–9]. Targeted inhibitors of eIF4A include two structurally related compounds, silvestrol and rocaglamide A (rocA), which inhibit translation by promoting the formation of stable eIF4A:RNA complexes and thereby preferentially inhibit translation of mRNAs with highly structured 5ʹ-UTRs and/or polypurine sequences [4, 5, 10–14]. Since RNA translation is downstream of the targets for many current anti-cancer drugs, including kinase inhibitors or inhibitors of receptor signaling, targeted inhibition of RNA translation may circumvent compensatory cross-over of upstream signaling pathways commonly linked to therapy resistance [8].

Chronic lymphocytic leukemia (CLL) is the most common form of adult leukemia [15–17] and is associated with accumulation of malignant B lymphocytes in the blood, bone marrow and secondary lymphoid organs. There are two subsets of CLL, U-CLL and M-CLL. U-CLL cases express unmutated immunoglobulin heavy variable chains (*IGHV*) and have a worse prognosis compared to M-CLL cases that originate from B cells that have undergone the germinal center reaction and express mutated *IGHV*. The B-cell receptor (BCR) is a major driver of disease progression [15] in both subsets of CLL and a classifier in calculating disease risk [18]. Stimulation of surface IgM (sIgM)/BCR induces a range of malignancy-promoting responses including activation of signaling pathways via PI3K, NF-κB and ERK, and downstream effector responses including increased expression of MYC [19, 20] and MCL1 [21] to promote growth/proliferation and cell survival, respectively. Importantly, sIgM stimulation also increases levels of both global and *MYC* mRNA-specific translation in CLL cells [22].

We previously showed that stimulation of RNA translation following BCR activation on CLL cells was associated with profound reprogramming of the translational machinery, including increased expression of two core components of eIF4F, a complex that is recruited to the 5ʹ m7G cap, containing several components including; eIF4A, and the scaffold protein, eIF4G [22]. BCR stimulation also reduced expression of PDCD4, a negative regulator of eIF4A [22–24]. Although BCR stimulation also increased global mRNA translation in normal B cells, this was not associated with changes in expression of eIF4A, eIF4G or PDCD4, suggesting that reprogramming of the translation initiation machinery may be selective for CLL cells [22].

A previous study demonstrated that silvestrol reduced expression of MCL1 and induced apoptosis of CLL cells in vitro. Silvestrol also improved outcomes in an *Eµ-TCL1* mouse model [25]. These studies clearly support the potential use of eIF4Ai as therapeutic agents for CLL, but further mechanistic analysis is required. For example, this study did not directly investigate effects of silvestrol on mRNA translation per se. Moreover, experiments were performed in the absence of stimulation of the BCR, a key driver and therapeutic target for CLL. It is particularly important to investigate effects of eIF4A inhibition on BCR responses in CLL, since BCR signaling itself has a profound impact on the translation machinery [22] and BCR signaling strongly increases expression of MCL1 (and MYC) [19, 21, 22] and could, therefore, influence susceptibility to eIF4Ai-mediated inhibition.

Here we have carried out the first study investigating the effects of eIF4A inhibitors (eIF4Ai) on BCR-driven responses in CLL cells. Overall, our study provides important new insights into the regulation of mRNA translation downstream of the BCR, consequences of eIF4A inhibition in malignant B cells and supports the concept that translational inhibition may be an attractive strategy for treatment of these diseases.

## Materials and methods

### Patients and cells

Peripheral blood mononuclear cells (PBMCs) were collected from patients with CLL attending clinic at the Southampton General Hospital (Table S1). None of the patients received any (immuno)chemotherapy, steroids or ibrutinib for the 6 months prior to collection. The study was approved by the Institutional Review Boards at the University of Southampton (REC: H228/02/t). All patients provided written informed consent.

PBMCs were isolated by density gradient centrifugation and cryopreserved in fetal bovine serum (FBS) with 10% dimethylsulfoxide (DMSO). *IGHV* mutational status and, surface IgM, CD38 and ZAP70 expression were determined as described [26, 27]. The median proportion of CD5^+^CD19^+^ cells was 95% (range 64–99%). BCR (sIgM) signaling capacity was determined by measuring the percentage of cells with increased intracellular Ca^2+^ (iCa^2+^) following stimulation with soluble goat F(ab’)_2_ anti-IgM [27]. The samples selected for this study were all considered as anti-IgM signaling responsive using a cut-off of anti-IgM-induced iCa^2+^ flux in ≥ 5% of cells. PBMCs samples from healthy donors were processed as previously described [27] and cryopreserved.

Following cryopreservation, PBMCs were thawed and recovered for 1 h at 37 °C in complete RPMI1640 medium (supplemented with 10% FBS, 2 mM glutamine and 1% penicillin/streptomycin). Cell viability determined by trypan blue exclusion was ≥ 90% in all cases. For BCR stimulation, samples were incubated with goat F(ab’)_2_ anti-human IgM or control F(ab’)_2_ (Southern Biotech, Cambridge Biosciences, UK) that had been bound to Dynabeads (Invitrogen, Paisley, UK) as described [20, 28]. Silvestrol and rocA were from MedChem Express (Stockholm, Sweden) and Sigma (Poole, UK), respectively. With the exception of viability assays and for incubations less than 6 h, cells were additionally treated with the caspase inhibitor Q-VD-OPh (5 μM; Sigma) to minimize secondary events due to apoptosis. Actinomycin D (actD; Thermofisher) was used at 5 μg/ml and cycloheximide (Sigma) was used at 10 μg/ml.

### Translation analysis

mRNA translation was analyzed using O-propargyl-puromycin (OPP)-labeling and polysome profiling, as described [22]. For analysis of CLL samples, cells were stained with anti-CD5-PerCyP5.5 and anti-CD19-pacific blue antibodies (BD Biosciences) for 15 min on ice and OPP-labeling was quantified in CD5^+^CD19^+^ cells. For PBMCs from healthy donors, cells were stained with anti-CD5-APC-Cy7, anti-CD19-pacific blue, anti-CD27-PerCP-Cy5.5 and anti-IgG-FITC antibodies (all Biolegend) for 15 min on ice, and OPP-labeling was quantified separately on CD5^−^CD19^+^IgG^−^CD27^−^ and CD5^−^CD19^+^IgG^−^CD27^+^ cells. Polysome profiling was performed as described [22].

### Quantitative polymerase chain reaction (Q-PCR)

Total mRNA or fractions from polysome profiling were isolated using the Promega RNA extraction kit (Promega, Southampton, UK). cDNA synthesis was performed using MMLV reverse transcriptase and oligo-dT primers (both Promega, Southampton, UK). *MYC, MCL1* and *B2M* mRNA expression was quantified by Q-PCR using probes Hs00153408_m1, Hs00172036_m1 and Hs00984230_m1, respectively (Life Technologies). mRNA abundance was determined for each mRNA against a standard curve, providing cDNA values and relative mRNA expression was calculated by normalizing the obtained values against *B2M* mRNA.

### Annexin V/propidium iodide staining

Cells (1 × 10^6^) were washed in PBS and resuspended in 300 µl of annexin V staining buffer (10 mM HEPES HCl (pH 7.4), 140 mM NaCl, 2.5 mM CaCl_2_) supplemented with 2.5 μg/ml fluorescein isothiocyanate-labeled annexin V (Protein Core Facility, University of Southampton) and 12.5 µM propidium iodide (Invitrogen). Cells were incubated in the dark for 15 min before analysis by flow cytometry (Canto II system, BD Biosciences). Unstained cells were used to set gates for identification of annexin V/propidium iodide negative and positive cells.

### Immunoblot analysis

SDS–PAGE was performed using equal protein loading following quantitation of protein content using the BioRad Protein Assay (BioRad, Hemel Hempstead, UK). Immunoblot analysis was performed using the following antibodies; anti-T^202^/Y^204^-phosphorylated ERK1/2, anti-ERK1/2, (both Cell Signaling Technology, Hitchin, UK), anti-MYC (9E10; Calbiochem), anti-MCL-1 (Santa Cruz Biotechnology), anti-GAPDH (Cell Signaling Technologies) and anti-HSC70 (Santa Cruz, Heidelberg, Germany). Secondary antibodies were HRP-conjugated rabbit, mouse or goat antibodies (GE Healthcare, Amersham, UK) and images were captured using the ChemiDoc-It Imaging System with a BioChemi HR camera (UVP, Cambridge, UK). Immunoblot signals were quantified using ImageJ (http://imagej.nih.gov/ij/). Expression of phospho-ERK1/2 was normalized to total ERK1/2 expression, whereas expression of MYC and MCL1 were normalized to loading controls (GAPDH or HSC70).

### Statistics

Statistical comparisons were performed using Prism 6 software (GraphPad Software, La Jolla, CA, USA).

## Results

### eIF4Ai reduces anti-IgM-induced global mRNA translation in CLL cells independent of changes in viability

**Fig. 1:**
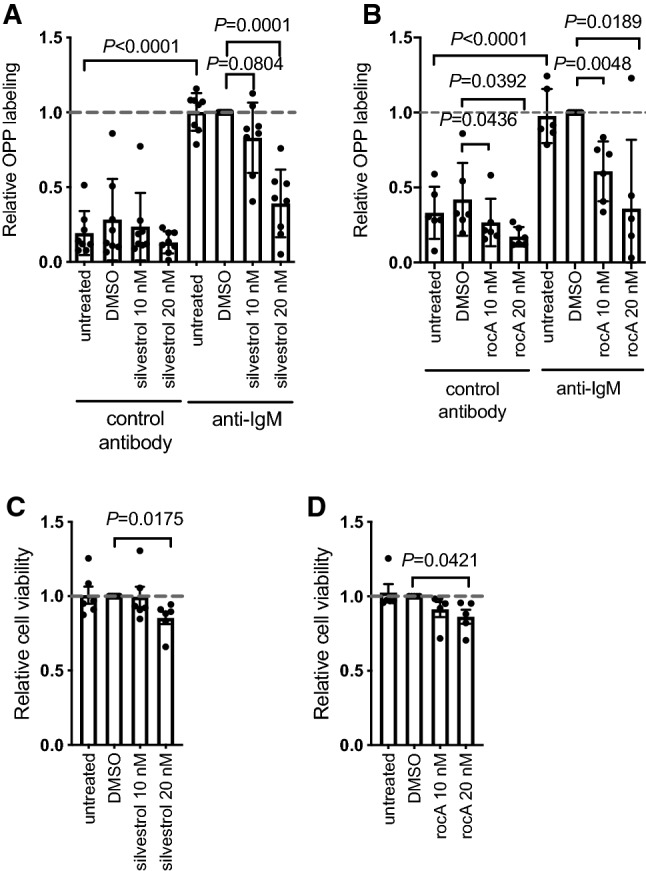
Effect of eIF4Ai on basal and anti-IgM-induced global mRNA translation. **A**, **B** CLL samples were pre-treated with (**A**) silvestrol (*n* = 8) or (**B**) rocA (*n* = 6), with DMSO or left untreated as a control for 1 h and then incubated with control antibody or anti-IgM for an additional 24 h. mRNA translation was quantified in CD19^+^CD5^+^ CLL cells using OPP-labeling. Graphs show values for individual samples and mean (± SD) OPP-labeling with values for DMSO/anti-IgM-treated cells set to 1.0. Student’s *t* tests were performed to determine the statistical significance of changes induced by anti-IgM alone, and DMSO or silvestrol in the presence of control antibody or anti-IgM (where not shown *P* > 0.05). **C**, **D** CLL samples were treated with the indicated concentration of (**C**) silvestrol (*n* = 6) or (**D**) rocA (*n* = 5), with DMSO or left untreated for 24 h, in the absence of Q-VD-OPh, prior to analysis of cell viability by annexinV/PI staining. Graphs show values for individual samples and mean (± SD) viable (annexinV^−^/PI^−^) cells with values for DMSO-treated cells set to 1. Student’s *t* tests were performed to determine the statistical significance of differences between test conditions and DMSO-treated cells (where not shown, *P* > 0.05)

We investigated the effect of silvestrol and rocA on global mRNA translation in CLL cells in the presence or absence of anti-IgM. Global mRNA translation was analyzed by labeling cells with the puromycin analogue OPP followed by flow cytometry allowing us to quantify OPP-labeling selectively within the CD19^+^CD5^+^ population of malignant cells in the patient blood samples. The inhibitors were tested at 10 and 20 nM, in line with previous publications [8, 25, 29]. All samples were considered as anti-IgM responsive based on analysis of intracellular calcium fluxes [27].There were low levels of basal mRNA translation in unstimulated cells and this was significantly increased following anti-IgM treatment (Fig. 1A, B). Silvestrol and rocA both caused a dose-dependent reduction of the induction of OPP-labeling in anti-IgM-treated cells (Fig. 1A, B). This was statistically significant for both compounds at 20 nM, but only for rocA at 10 nM. At 20 nM, rocA reduced OPP-labeling in anti-IgM-treated cells to a level very similar to that of unstimulated cells, whereas, on average, the inhibitory effects of silvestrol at 20 nM on anti-IgM-induced OPP-labeling were somewhat less complete. There was also significant variation in the degree of inhibition of OPP-labeling by eIF4Ai between samples (Fig. 1A, B). Since the samples analyzed were selected based on competence to signal in response to anti-IgM, it was not meaningful to determine whether this variation in response to eIF4Ai was related to clinical features, such as *IGHV* mutation status, and ZAP70 or CD38 expression, as these features are themselves closely correlated with signal capacity [27]. There was no clear correlation between anti-IgM signaling strength and the degree of inhibition of OPP-labeling by silvestrol or rocA (Supplementary Fig. 1).

Effects of silvestrol on translation were also analyzed using polysome profiling (Supplementary Fig. 2). As previously reported [22], anti-IgM increased the abundance of polysomes compared to control antibody-treated cells. This was reversed by silvestrol (but not DMSO) which generally decreased polysome peak amplitude and increased accumulation of RNA in monosome peaks. However, the profiles also confirmed responses varied between samples. For example, in sample 523 10 nM silvestrol resulted in accumulation in both 60S and 80S, while for sample 575, 10 nM silvestrol only decreased polysome peaks. Moreover, for this sample, 20 nM silvestrol substantially diminished polysome peaks while 20 nM silvestrol appeared to have little effect in sample 654.

Although silvestrol induces apoptosis in CLL cells with an IC_50_ of ~ 10 nM following 72 h of drug exposure [25], it seemed unlikely that the inhibitory effects of eIF4Ai on mRNA translation were a consequence of reduced viability since our experiments were performed at an earlier time point (24 h), in the presence of the caspase inhibitor, Q-VD-OPh, and the cells analyzed for OPP labeling were gated on the viable cell population according to their FFC/SSC. However, to address directly the possibility that decreased mRNA translation could have been a consequence of decrease cell viability, we investigated the effects of eIF4Ai on the spontaneous apoptosis that occurs when CLL cells are placed in culture using annexin V/PI staining (without Q-VD-OPh). eIF4Ai only very modestly increased apoptosis at the highest concentration tested (20 nM) at 24 h (Fig. 1C and D).

### Characterisation of mRNA translation and effect of silvestrol in normal B cells

In our previous study, we demonstrated that although anti-IgM increased global mRNA translation in normal B cells, this was not associated with the changes in expression of eIF4A or PDCD4 that were observed in CLL cells [22]. We, therefore, investigated the effects of eIF4Ai on mRNA translation in normal B cells. PBMCs from healthy donors contain a heterogeneous mix of B cells and it was important to determine whether induction of mRNA translation by anti-IgM, or potential inhibitory effects of eIF4Ai, differed between subsets. We excluded IgG positive cells from the analysis (since these would not be able to respond to anti-IgM stimulation) and gated on CD27-negative or positive cells to identify naive and memory B cells, respectively (Supplementary Fig. 3A). The populations studied, therefore, largely reflected naive (IgM^+^CD27^−^) and non-switched memory (IgM^+^CD27^+^) B cells, likely counterparts of U-CLL and M-CLL, respectively [30, 31]. Stimulation was performed using soluble F(ab’)_2_ anti-IgM which is more effective than bead-bound anti-IgM for activation of mRNA translation in normal B cells [22].

**Fig. 2:**
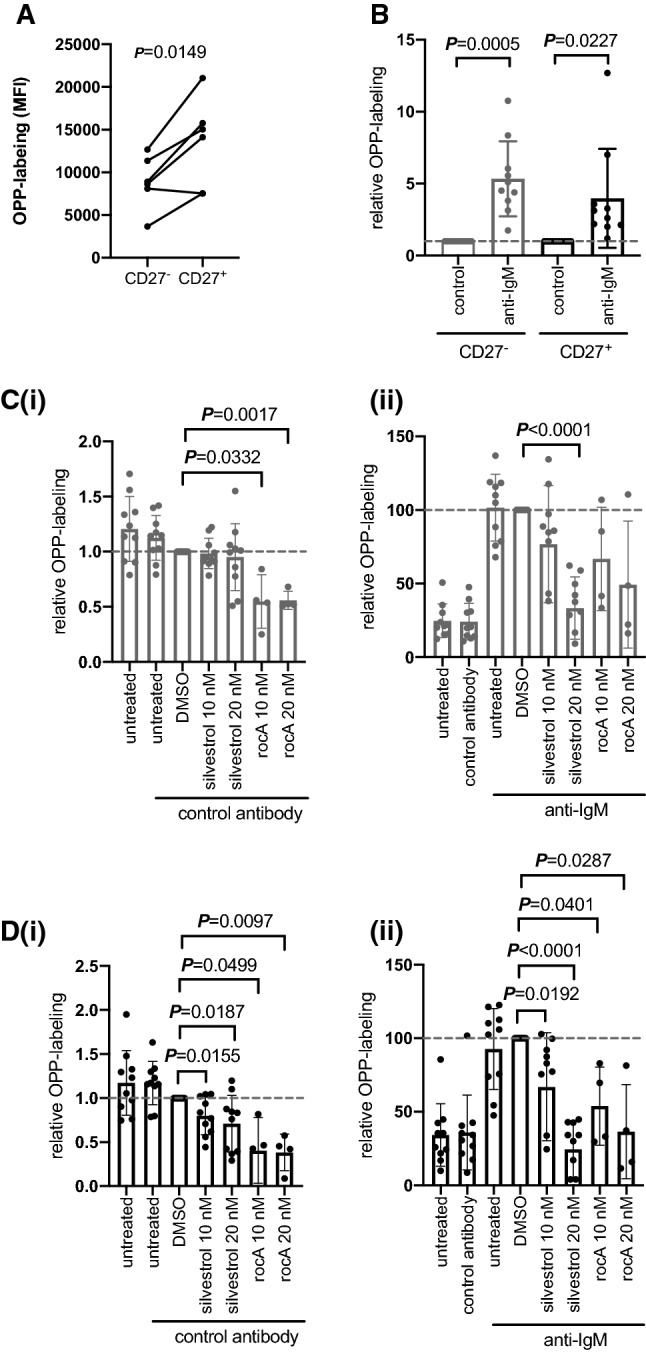
Effect of eIF4Ai on basal and anti-IgM-induced global mRNA translation in normal B cells. **A** PBMCs from healthy donors (*n* = 10) were incubated for 24 h prior to OPP labeling. OPP levels were quantified in CD19+ CD5− IgG− cells and then on gated CD27− or CD27+ sub-populations. Each dot and adjoining line represents an individual donor’s cells. **B** PBMCs from healthy donors were stimulated with soluble anti-IgM or control antibody for 24 h prior to OPP labeling. Graphs show values for individual samples and mean (± SD) OPP-labeling with values for control antibody-treated cells set to 1.0. **C**, **D** PBMCs from healthy donors were pre-treated with silvestrol (*n* = 10), rocA (*n* = 4) or DMSO or left untreated as a control for 1 h and then incubated with control antibody (**i**) or soluble anti-IgM (**ii**) for an additional 24 h. mRNA translation was quantified in CD19+ CD5− IgG− CD27− (**C**), or CD19+ CD5− IgG− CD27 + (**D**) cells. Graphs show values for individual samples and mean (± SD) OPP-labeling with values for DMSO treated cells set to 1.0. **A**–**D** Student’s *t* tests were performed to determine the statistical significance of changes (where not shown *P* > 0.05), *n* = 6

Consistent with previous studies [30], CD27^−^ cells were more abundant than CD27^+^ cells amongst IgG^−^CD19^+^ cells, although the relative proportions of CD27^±^ cells varied considerably between donors (Supplementary Fig. 3B). The basal translation levels also varied between these two subsets as CD27^+^ B cells had significantly higher basal OPP-labeling compared to CD27^−^ cells (Fig. 2A). OPP labeling was enhanced in both subsets following anti-IgM treatment compared to control antibody treatment (Fig. 2B).

Next, we analyzed the effect of eIF4Ai on translation. Silvestrol had no effect on OPP-labeling in unstimulated CD27^−^ cells, while rocA reduced levels by ~ 50% (Fig. 2C(i)). By contrast, both eIF4Ai significantly reduced the elevated levels of basal translation in CD27^+^ cells, although rocA had a greater effect than silvestrol (Fig. 2D(i)). Although both eIF4Ai reduced anti-IgM-induced translation in either cell population, these effects were significant for both compounds at 10 and 20 nM in CD27^+^ cells, but only for silvestrol at 20 nM in CD27^−^ cells (Fig. 2C(ii) and D(ii)).

Overall, these data demonstrate that non-switched memory B cells have higher basal translation levels compared to naïve B cells. Increased basal translation levels may mean that memory B cells are primed for restimulation and facilitate subsequent antibody synthesis (which requires very large RNA translation capacity). However, translation can be enhanced in both subsets following sIgM stimulation. Translation in CD27^+^ cells appeared to be most susceptible to eIF4Ai, as both inhibitors reduced basal and induced translation at either concentration. Interestingly, rocA appeared to be more active against basal translation in both CD27^−^ and CD27^+^ cells, pointing to potential differences in the mechanisms of action of these compounds.

### eIF4Ai reduced anti-IgM-induced MYC protein expression but profoundly increased *MYC* mRNA in CLL cells

**Fig. 3:**
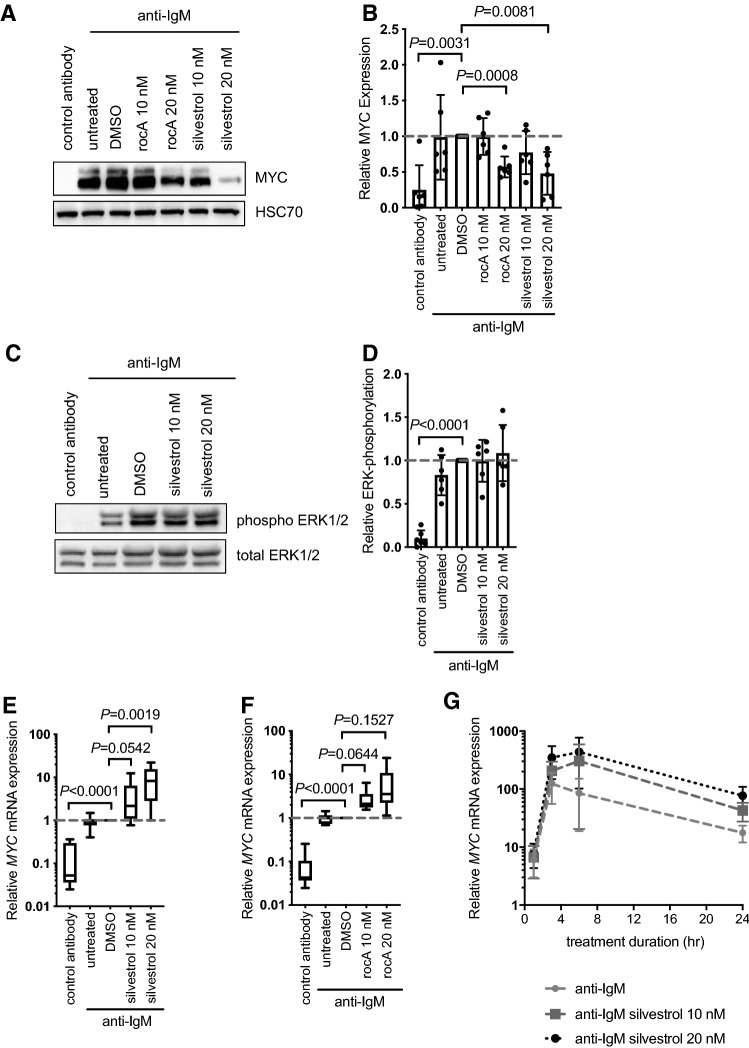
Effect of eIF4Ai on anti-IgM-induced MYC protein and mRNA expression. CLL samples were pre-treated with silvestrol, rocA or DMSO (solvent control) or left untreated for 1 h and then incubated with anti-IgM for 6 h. Samples were also treated with control antibody for 6 h. Expression of (**A**, **B**) MYC and HSC70 (loading control) and (**C**, **D**) total and phosphorylated ERK1/2 was analyzed by immunoblotting. Figures show **A**, **C** representative immunoblots and **B**, **D** quantification for all samples studied (*n* = 6). Expression of *MYC* mRNA was quantified following 24 h treatment using Q-PCR for (**E**) silvestrol or (**F**) rocA-treated cells. Graphs show mean (± SD) relative *MYC* expression (normalized against *B2M*), with values for DMSO/anti-IgM-treated cells set to 1.0. Student’s *t* tests were performed to determine the statistical significance of differences between test conditions and DMSO/anti-IgM-treated cells (where not shown, *P* > 0.05). **G** CLL samples (*n* = 4) were pre-treated with silvestrol, DMSO or left untreated for 1 h prior to incubation for 1, 3, 6 or 24 h. Expression of *MYC* mRNA was quantified using Q-PCR. Graph show mean expression with values for control antibody treated cells set to 1.0 for each time point

We next investigated effects of eIF4Ai on anti-IgM-induced MYC protein expression in CLL cells. MYC was analyzed at 6 h post-stimulation in line with previous studies demonstrating that MYC induction was associated with both increased transcription and translation [19, 22]. Immunoblot analysis demonstrated that eIF4Ai significantly inhibited anti-IgM-induced MYC expression although silvestrol appeared somewhat more effective than rocA (Fig. 3A, B).

To assess whether eIF4Ai affected kinase pathways activated directly downstream of sIgM, phosphorylation of ERK1/2 was analyzed. Silvestrol had no effect on anti-IgM-induced ERK1/2 phosphorylation at 24 h post-stimulation, demonstrating that inhibitory effects on MYC expression were not due to effects on upstream signaling (Fig. 3C, D). Anti-IgM-induced ERK1/2 phosphorylation was similarly unaffected by rocA (data not shown). We also investigated effects on phosphorylation of AKT and p38-MAPK. These were analyzed at 3 h post-stimulation since, in contrast to ERK1/2, AKT phosphorylation is transient following stimulation with anti-IgM beads. However, neither silvestrol nor rocA had any effect on phosphorylation of AKT or p38-MAPK (Supplementary Fig. 4). Taken together, these results show that inhibition of anti-IgM-induced MYC expression by eIF4Ai was not due to inhibition of sIgM signaling per se*.*

To investigate the mechanism of inhibition of MYC protein expression, we analyzed the effects of eIF4Ai on the expression of *MYC* mRNA. Although silvestrol did not significantly alter *MYC* mRNA expression in the absence of sIgM stimulation (Supplementary Fig. 5A), the combination of silvestrol and anti-IgM caused a striking accumulation of *MYC* mRNA compared to anti-IgM alone (Fig. 3E). Thus, whereas anti-IgM alone increased *MYC* mRNA expression by ~ tenfold, the combination of anti-IgM and silvestrol resulted in an ~ 70-fold increase in *MYC* mRNA expression compared to unstimulated cells. This apparent accumulation of *MYC* mRNA was not a consequence of reduced expression of housekeeping *B2M* mRNA (used to normalize *MYC* mRNA) as *B2M* mRNA was unaffected by anti-IgM in the presence or absence of silvestrol (Supplementary Fig. 5B). Similar effects of eIF4Ai on anti-IgM-induced *MYC/B2M* mRNA expression were obtained using rocA (Fig. 3F and Supplementary Fig. 5B).

We performed detailed time-course experiments to characterize the kinetics of this unexpected accumulation of *MYC* mRNA in anti-IgM/silvestrol-treated cells (Fig. 3G). Induction of *MYC* mRNA in anti-IgM-only treated cells was detected at 1 h following stimulation, this was further increased at 6 h and maintained at a relatively high level for 24 h. The enhancing effects of silvestrol were first apparent at 3 h post-stimulation and were maintained up to 24 h.

Therefore, inhibition of anti-IgM-induced MYC protein expression in cells exposed to eIF4Ai was not a consequence of reduced *MYC* mRNA expression. In fact, the combination of anti-IgM and eIF4Ai caused an unexpected accumulation of *MYC* mRNA.

### Silvestrol enhances *MYC* mRNA stability

The ability of eIF4Ai to further induce *MYC* mRNA levels in the presence of anti-IgM was unexpected. Previous studies demonstrated tight linkage between *MYC* mRNA translation and degradation. Thus, inhibition of translational elongation using CHX decreases *MYC* mRNA degradation such that *MYC* mRNA accumulates in CHX-treated cells [32–34]. We, therefore, investigated whether accumulation of *MYC* mRNA following inhibition of translational initiation by eIF4Ai in anti-IgM-treated cells was also associated with *MYC* mRNA stabilization.

mRNA stability was investigated by treating cells with the RNA polII inhibitor actinomycin D (actD) to prevent de novo synthesis and quantifying the decline in *MYC* mRNA expression. In initial experiments, we demonstrated that *MYC* mRNA had a short half-life (< 30 min) in the presence or absence of anti-IgM (Supplementary Fig. 6) consistent with the rapid turnover of *MYC* mRNA observed in many other systems [35, 36]. AnnexinV/PI staining demonstrated that actD did not induce apoptosis over this time course (data not shown).

**Fig. 4:**
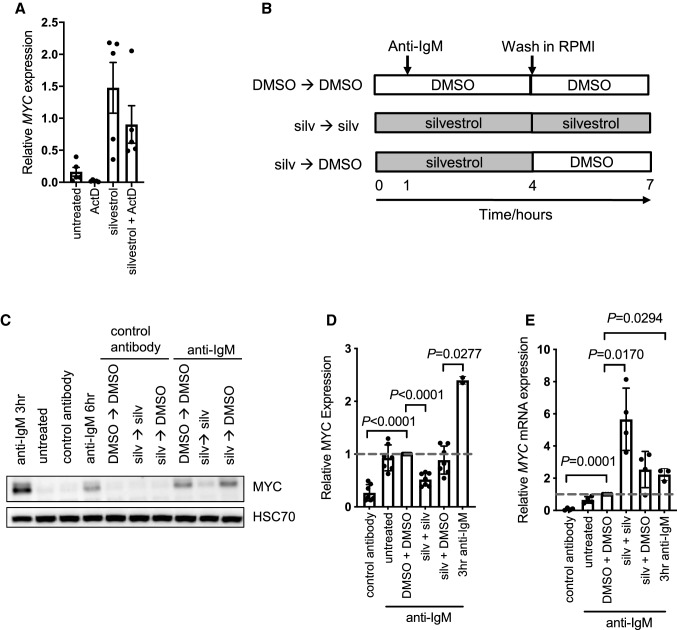
Effect of silvestrol on *MYC* mRNA stability and protein expression following removal of silvestrol. **A** CLL samples (*n* = 5) were pre-treated with silvestrol (20 nM) or left untreated for 1 h prior to 24 h incubation with anti-IgM. Cells were then treated with or without actinomycin D for an additional hour. *MYC* mRNA was quantified using Q-PCR. Graph shows values for individual samples and mean (± SD) MYC expression (relative to *B2M*). **B** Schematic of wash-out experiments. **C**–**E** CLL cells were pre-treated with silvestrol (20 nM) or DMSO, or left untreated for 1 h before addition of anti-IgM or control antibody. After 3 h, cells were washed thoroughly before addition of DMSO or re-addition of silvestrol (20 nM). Cells were then incubated for a further 3 h. Additionally samples were collected after 3 h in the presence of anti-IgM, as a control. MYC and HSC70 expression was analyzed by immunoblotting and *MYC* mRNA was quantified using Q-PCR. Figure shows (**C**) representative immunoblotting results and (**D**) quantitation of MYC protein (n = 4) and (**E**) mRNA (*n* = 7) for all samples analyzed. Graphs show values for individual samples and mean (± SD) expression with values for DMSO/anti-IgM-treated cells set to 1.0. Student’s *t* tests were performed to determine the statistical significance of differences between test conditions and continuous DMSO/anti-IgM-treated cells (where not shown, *P* > 0.05)

To investigate the effect of eIF4Ai on *MYC* mRNA stability following anti-IgM stimulation, cells were either pre-treated with silvestrol for an hour, or left untreated as a control, and stimulated with anti-IgM for a further 24 h. Cells were then treated with actD or left untreated for an additional hour and *MYC* mRNA was quantified by Q-PCR (Fig. 4A). ActD reduced *MYC* levels in anti-IgM treated cells by ~ 90%, while actD reduced *MYC* mRNA by ~ 50% in cells treated with anti-IgM and silvestrol. Thus, *MYC* mRNA is stabilized in the presence of silvestrol. To assess the translational efficiency of *MYC* RNA, we carried out qPCR analysis from polysome profile fractions for control cells, and cells treated with anti-IgM in the presence of DMSO or silvestrol (20 nM) (Supplementary Fig. 7A). In sample 575, there was a clear shift in the distribution of *MYC* mRNA to higher fractions in anti-IgM-treated cells compared to controls, and this shift was effectively reversed by silvestrol. There was also a rightward shift in the distribution of *MYC* mRNA with anti-IgM alone, compared to DMSO, in sample 523 with an accumulation of *MYC* mRNA in fraction 7. In this sample, silvestrol resulted in a clear increase in the accumulation of *MYC* RNA in the monosome peak (fraction 4) and loss of *MYC* mRNA in fractions 5 and 6 (Supplementary Fig. 7B). However, the fraction 7-associated RNA seemed relatively unaffected by silvestrol. These results indicate that, overall, anti-IgM increases *MYC* RNA translation, and this is reduced by silvestrol. However, similar to analysis of polysome distribution (Supplementary Fig. 2), there was substantial variation between samples. Indeed, in sample 654, there was not a clear rightward shift for *MYC* mRNA distribution in anti-IgM-treated cells, or a clear inhibitory effect of silvestrol (Supplementary Fig. 7C). This indicated that this sample had high basal *MYC* translation that was not enhanced with anti-IgM treatment. Interestingly, this sample did not show sensitivity to silvestrol at 10 or 20 nM via polysome profiling (Supplementary Fig. 2C) thus the lack of increased *MYC* translation in response to anti-IgM and lack of inhibition in response to silvestrol treatment highlights the variable nature of CLL response as seen in Fig. 1A.

### Silvestrol-induced *MYC* mRNA accumulation is reversible

It was important to determine the fate of the accumulated *MYC* mRNA and we, therefore, performed wash-out experiments to determine whether removal of eIF4Ai was associated with a strong “burst” of MYC protein expression from the accumulated mRNA pool or whether the accumulated *MYC* mRNA remained blocked for translation. CLL cells were pretreated with silvestrol or DMSO for 1 h and then exposed to control antibody or anti-IgM, we used silvestrol at 20 nM as this concentration resulted in significant accumulation in *MYC* mRNA, with only a small decrease (17%) in cell viability (Fig. 1C). Moreover, the experiment was performed with addition of Q-VD-OPh to suppress apoptosis. After 3 h, cells were washed extensively to remove drug. Cells were then incubated for a further 3 h with silvestrol (continuous exposure) or with DMSO (wash-out). MYC expression was quantified after a further 3 h giving a total anti-IgM exposure of 6 h (Fig. 4B).

As expected, anti-IgM alone for 6 h resulted in significant increase in MYC protein (Fig. 4C, D). The shorter-time point of 3 h resulted in greater MYC protein induction, demonstrating that this protein is rapidly expressed in response to anti-IgM (Fig. 4C, D). Moreover, MYC was not induced in cells exposed to control antibody under any condition and MYC induction in anti-IgM-treated cells was substantially reduced by the continued presence of silvestrol. Anti-IgM-induced MYC expression was also not affected by continuous exposure to DMSO demonstrating that the additional washes and cell manipulations did not affect MYC induction. Importantly, the amount of MYC induced by anti-IgM in cells exposed to silvestrol for 3 h followed by DMSO was similar to that in cells continuously exposed to DMSO. Parallel analysis of *MYC* mRNA confirmed that continual exposure to silvestrol increased *MYC* mRNA levels in cells treated with anti-IgM and demonstrated that following silvestrol wash-out *MYC* mRNA levels declined (Fig. 4E). Removal of silvestrol after 3 h (of the 6 h anti-IgM treatment) resulted in *MYC* mRNA levels similar to anti-IgM levels following 3 h incubation. Taken together these data demonstrate that the silvestrol induced accumulation of *MYC* mRNA seen after 6 and 24 h (Fig. 3F and 4E) can be reversed upon silvestrol removal and was not translated into protein as there was significantly less protein expression following silvestrol removal compared to 3 h anti-IgM treatment (Fig. 4C, D).

Overall, these data indicate that effects of silvestrol on *MYC* mRNA accumulation in anti-IgM-treated cells were reversible since *MYC* mRNA levels declined following drug washout, presumably as a consequence of increased mRNA turnover following release of eIF4Ai-mediated translational blockade. Importantly, the silvestrol-induced accumulated *MYC* mRNA does not appear to be competent to re-enter translation since drug removal is not associated with a burst of MYC expression.

### eIF4Ai inhibited anti-IgM-induced *MCL1* translation without substantial mRNA accumulation

**Fig. 5:**
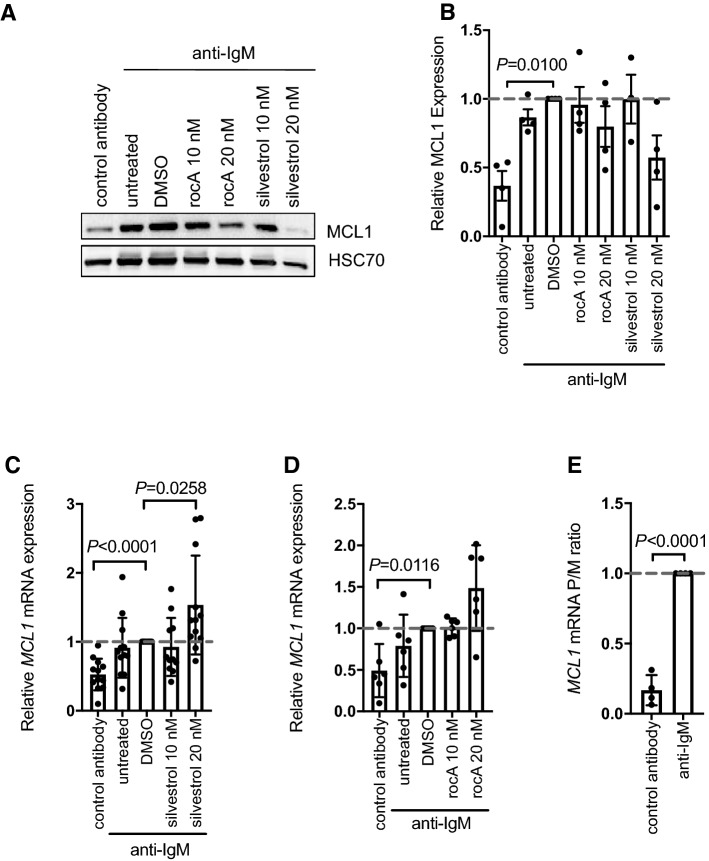
Effect of eIF4Ai on anti-IgM-induced MCL1 protein and mRNA expression. CLL samples were pre-treated with silvestrol, rocA or DMSO (solvent control) or left untreated for 1 h and then incubated with anti-IgM for 24 h. Samples were also treated with control antibody. After 24 h expression of (**A**, **B**) MCL1 and HSC70 (loading control) was analyzed by immunoblotting. Figure **A** shows representative immunoblot and (**B**) quantification of all samples analyzed (*n* = 4). **C**, **D** Expression of *MCL1* mRNA was quantified by Q-PCR for **c** silvestrol (*n* = 12) or **D** rocA-treated cells (*n* = 6). Student’s *t* tests were performed to determine the statistical significance of differences between test conditions and DMSO/anti-IgM-treated cells (where not shown, *P* > 0.05). **E** Polysome profiling of *MCL1* mRNA (*n* = 4). Graphs show values for individual samples and mean (± SD) expression of polysome/monosome (*P*/*M*) ratio with values for DMSO/anti-IgM treated cells set to 1.0. A Student’s *t* test was performed to determine the statistical significance of differences between control antibody and anti-IgM-treated cells

We next investigated effects of eIF4Ai on expression of MCL1 which is a major survival factor for CLL cells and, like MYC, is induced following sIgM stimulation [21]. MCL1 expression can be regulated at many levels, including via control transcription, translation and protein degradation, and it is currently unknown to what extent increased *MCL1* mRNA translation contributes to accumulation of MCL1 protein in anti-IgM-stimulated CLL cells. We, therefore, first investigated whether anti-IgM increased *MCL1* mRNA translation to determine whether MCL1 was a bone fide target for translational control in CLL cells. Analysis was performed at 24 h after stimulation, in line with the original study showing anti-IgM-induced MCL1 expression [21]. Anti-IgM caused an ~ threefold increase in MCL1 protein expression (Fig. 5A, B) and increased *MCL1* mRNA levels by ~ twofold (Fig. 5C, D). Polysome profiling revealed a clear increase in the polysome/monosome ratio for *MCL1* mRNA, indicating translational induction (Fig. 5E). Thus, like *MYC*, *MCL1* is a target for translational control downstream of the BCR in CLL cells.

Immunoblot analysis demonstrated that silvestrol significantly reduced accumulation of MCL1 protein in anti-IgM-treated cells (Fig. 5A,B). Similar results were obtained with rocA, although overall effects were more modest, when compared to MYC. However, in marked contrast to *MYC*, eIF4Ai only very modestly (~ 50%) increased *MCL1* mRNA levels in anti-IgM-treated cells (Fig. 5C, D).

Thus, eIF4Ai deprive CLL cells of both growth/proliferation (MYC) and survival (MCL1) effector arms of BCR signaling. However, only for MYC was there a striking increase in mRNA.

## Discussion

Despite the success of kinase inhibitors (such as ibrutinib and idelalisib), BCL2 inhibitors (e.g., venetoclax) and anti-CD20 monoclonal antibodies in the treatment of some B-cell cancers, there is a pressing need for new therapeutic strategies for these diseases due to resistance and/or poor response. Clinical development of mRNA translation inhibitors is currently focused on inhibition of upstream mRNA translation promoting pathways (such as signaling mediated by mTORC1 and MNKs) or inhibition of eIFs and translation elongation factors [6]. New translation inhibitors have the potential to be combined with current treatment regimens to enhance response by targeting a discrete downstream effector function of BCR signaling as part of a potential multi-hit treatment approach. By preventing the expression of key downstream effectors of survival and proliferation, translational inhibition may avoid mechanisms that commonly mediate resistance to kinase inhibitors, such as acquisition/selection of mutations within the targeted pathways or “rewiring” of signaling to circumvent the pharmacological blockade [8, 37]. Indeed, a recent publication using MYC-driven lymphoma cell lines also demonstrated that eIF4Ai was superior to mTOR inhibitors for inhibiting MYC expression [9]. Here, we demonstrate that eIF4Ai deprive CLL cells of both survival and growth promoting effectors induced following BCR activation and provide important new insight into the mechanisms of action of these compounds.

Our experiments demonstrated that eIF4Ai have relatively little effect on apoptosis at 24 h at concentrations up to 20 nM (17 and 18% decrease with silvestrol and rocA, respectively). This observation, combined with the fact that we used Q-VD-OPh to prevent apoptosis in OPP-labeling and immunoblotting experiments, demonstrates that the reduction in mRNA translation and MYC/MCL1 expression induced by eIF4Ai is not a secondary consequence of apoptosis. However, it is important to stress that prolonged exposure to silvestrol does effectively induce CLL cell death [25, 38]. For example, Lucas et al. demonstrated that silvestrol induced CLL cell death with an IC_50_ of 6.9 nM when assayed after 72 h continuous drug exposure [25]. Thus, the relatively rapid effects of silvestrol on protein expression, including down-modulation of the survival protein MCL1, are followed by cell killing, and this likely drives the therapeutic efficacy of silvestrol observed in mouse models [25].

Pharmacological inhibition of eIF4A was sufficient to reduce anti-IgM-induced mRNA translation indicating that eIF4A is a critical node linking BCR signaling (potentially via increased expression of eIF4A itself, and reduced expression of its inhibitor PDCD4 [22]) to control of mRNA translation. Importantly, MYC has been shown to promote expression of various components of the translational machinery, including eIF4A, suggesting the operation of a feed-forward loop in stimulated CLL cells whereby eIF4A facilitates MYC production which in turn enhances eIF4A expression [39]. Thus, in a similar mechanism to that described by Wiegering et al. in colorectal cancer, inhibition of eIF4A could preferentially inhibit BCR-induced translation in CLL [8]. We focused on MYC, due to this potential feed-forward mechanism as well as its likely clinical importance as a powerful driver of proliferation and cell growth downstream of the BCR [19, 22]. We also focused on the pro-survival protein, MCL1, a known BCR-target that has been shown to correlate with therapy resistance in CLL [25].

Our results clearly demonstrate that eIF4Ai reduce expression both MYC and MCL1 following sIgM stimulation and are, therefore, a potentially powerful approach to counter proliferation and survival-promoting responses. However, it is important to recognize that MYC and MCL1 are unlikely to be the only targets regulated translationally by BCR signaling in an eIF4A-dependent manner and profiling experiments have identified 100 s of silvestrol-sensitive RNAs in other cell systems [4, 5]. It was also notable that although silvestrol substantially reduced anti-IgM-induced OPP-labeling in CLL cells, there appeared to have been a small proportion of the induced translation that was resistant to silvestrol. Thus, although a large proportion of the induced translation appeared to be mediated by eIF4A (consistent with induction of eIF4A expression and reduced expression of PDCD4 in anti-IgM-treated cells [22]), translation of eIF4A-independent RNAs may also be induced following sIgM activation.

We used two structurally related compounds, silvestrol and rocA to directly investigate the function of eIF4A. The structural similarities between rocA and silvestrol may indicate that the two compounds work via a similar mechanism although the different responses seen with rocA treatment could be a consequence of rocA clamping onto eIF4A and limiting eIF4A for other eIF4F complexes [40, 41]. It has previously been demonstrated that rocA inhibits translation of specific mRNAs by increasing the affinity of eIF4A to polypurine sequences in the 5ʹ UTR, independently of ATP [13]. This rocA-eIF4A complex prevents the pre-initiation complex (PIC) scanning the mRNA to initiate translation but it has been demonstrated that rocA can also inhibit internal ribosome entry site translation [13]. Interestingly, our results point to some subtle differences in responses to these compounds which may reflect differences in action and/or potency. For example, rocA had a greater effect on global mRNA translation levels in CLL cells (with complete reversal of anti-IgM-induced translation) suggesting that silvestrol resistant mRNAs could be inhibited by the dual action of rocA. RocA also more effectively inhibited basal translation in normal B cell subsets, whereas silvestrol appeared to be somewhat more effective for inhibition of MYC induction in CLL cells.

This project focused on the impact of eIF4Ai in CLL cells, therefore, we compared our primary samples to sIgM naïve (CD27−) or non-switched IgM memory (CD27+) B cells, that are predicted to be the cell of origin for unmutated (U) or mutated (M) CLL, respectively. We demonstrate the non-switched IgM memory B cells had higher basal translation compared to naïve B cells, and non-switched IgM memory B cells were susceptible to eIF4Ai. Both subsets were sensitive to silvestrol repression of anti-IgM-induced translation despite eIF4A levels being unaltered in response to anti-IgM treatment [22]. Anti-IgM signals via mTORC1 which is known to regulate mRNA translation. mTORC1 activates p70S6K that can enhance the helicase activity of eIF4A via phosphorylation of eIF4B and PDCD4, leading to eIF4B interacting with eIF4A and degradation of PDCD4, the negative regulator of eIF4A [42]. Thus the unchanged levels of eIF4A in response to anti-IgM stimulation seen in healthy donor B cells [22] may not indicate activity level of eIF4A, thus silvestrol was able to inhibit translation in these cells when eIF4A levels were unaltered. It is also believed that eIF4A may play a helicase-independent role in global translation possibly due to remodeling of the 40S ribosomal subunit [43].

This ability of silvestrol to significantly alter *MYC* mRNA may provide a mechanism for selectivity in vivo, with malignant cells being more dependent on MYC expression as a driver of disease progression*.* Silvestrol has been shown to have a selective apoptotic effect on malignant cells compared to healthy B cells [25], therefore, the particularly striking accumulation of *MYC* mRNA alteration may highlight the effectiveness of silvestrol specifically in MYC-driven tumors. The stabilization of *MYC* mRNA in response to silvestrol was not seen with *MCL1* mRNA possibly due to the presence of stabilization elements within the *MYC* mRNA. Additionally, *MYC* mRNA has previously been showed to be stabilized by translation elongation inhibitors [32–34]. Our results suggest that inhibition of *MYC* mRNA translation by eIF4Ai may similarly prevent *MYC* mRNA turnover, but further studies will be required to more fully understand the mechanisms of this and its interplay, if any, with known *MYC* mRNA destabilization elements. Zhang et al. recently demonstrated that in lymphoma cell lines silvestrol treatment significantly enhanced eIF4A binding to *MYC* RNA using native RNA immunoprecipitation [9], which could provide a mechanism of stabilization of *MYC* RNA, but it is unclear whether this observed accumulation was as a consequence of the enhanced RNA levels as total *MYC* RNA levels were not published. Waldron et al. have demonstrated that inhibition of eIF4A by hippuristanol can alter the secondary structure of eIF4A-dependent mRNAs [43]. If this remodeling of secondary structure occurs with all eIF4Ai this could provide a mechanism for increased *MYC* mRNA stability seen here. Our wash-out experiments were performed to determine the fate of the accumulated *MYC* mRNA. This was particularly important since release of eIF4A inhibition in patients could result in a burst of MYC expression with potential tumor-promoting consequence. However, our in vitro experiments revealed that the fate of the accumulated mRNA appeared to be towards degradation, without translation, following removal of drug.

The effort to find selective eIF4A inhibitors is an exciting area of research [44] with novel compounds having similar effects. Peters et al. used target-based screening approach to identify the natural product elatol (isolated from the red alga *Laurencia microcladia*) as an inhibitor of eIF4A1 [38]. In vitro analysis demonstrated that elatol reduced global mRNA translation and reduced expression of MYC, cyclin D3, MCL1, BCL2 and PIM2 in DLBCL-derived cell lines. Like silvestrol, elatol also reduced induction of MYC in primary CLL cells following BCR stimulation and reduced the growth of B-lymphoma cells in vivo [38]. Chen et al. recently reported synthetic derivatives of pateamine A which were originally isolated from the sea sponge *Mycale* sp. and like silvestrol, appear to promote association between eIF4A and mRNA [45]. The derivative DMDAPatA induced apoptosis of CLL cells and synergized with ABT-199 to enhance cell killing. Recently, eFFECTOR Therapeutics’ new compound, eFT226, was shown to selectively inhibit translation of eIF4A selective genes that was dependent on the presence of the 5ʹ-UTR sequences for selectivity [46]. eFT226 also caused a G1 cell cycle arrest in lymphoma cell lines due to the inhibition of translation of MYC, CDK4 and cyclin D1, which led to anti-tumor activity in vivo [46]. Overall, our study demonstrates that eIF4Ai can deprive CLL cells of both pro-proliferation and pro-survival effectors following BCR activation and may be an effective therapeutic strategy for B-cell malignancies.

### Supplementary Information

Below is the link to the electronic supplementary material.

Supplementary file1 (PPTX 939 KB)

## Data Availability

All data generated or analyzed during this study are included in this published article [and its supplementary information files].
