# Supplementary material for: Targeted inhibition of eIF4A suppresses B-cell receptor-induced translation and expression of MYC and MCL1 in chronic lymphocytic leukemia cells
Source: Cell Mol Life Sci. 2021 Aug 16;78(17-18):6337–49. doi: 10.1007/s00018-021-03910-x (PMC8429177; doi:10.1007/s00018-021-03910-x)
Supplement: Supplementary file 1 — Supplementary file1 (PPTX 939 KB) [file 18_2021_3910_MOESM1_ESM.pptx]

## Slide 1
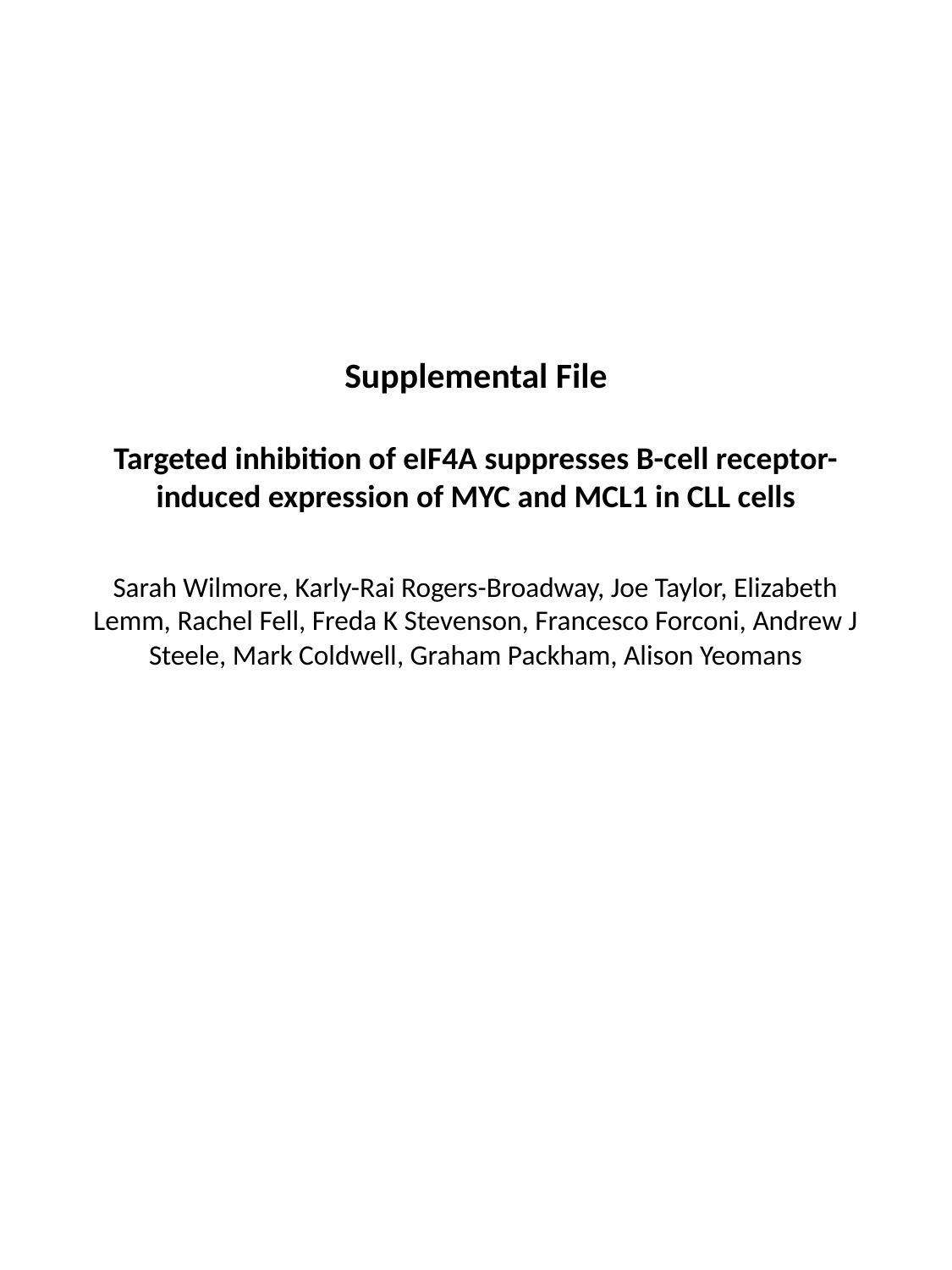

# Supplemental File Targeted inhibition of eIF4A suppresses B-cell receptor-induced expression of MYC and MCL1 in CLL cells Sarah Wilmore, Karly-Rai Rogers-Broadway, Joe Taylor, Elizabeth Lemm, Rachel Fell, Freda K Stevenson, Francesco Forconi, Andrew J Steele, Mark Coldwell, Graham Packham, Alison Yeomans

## Slide 2
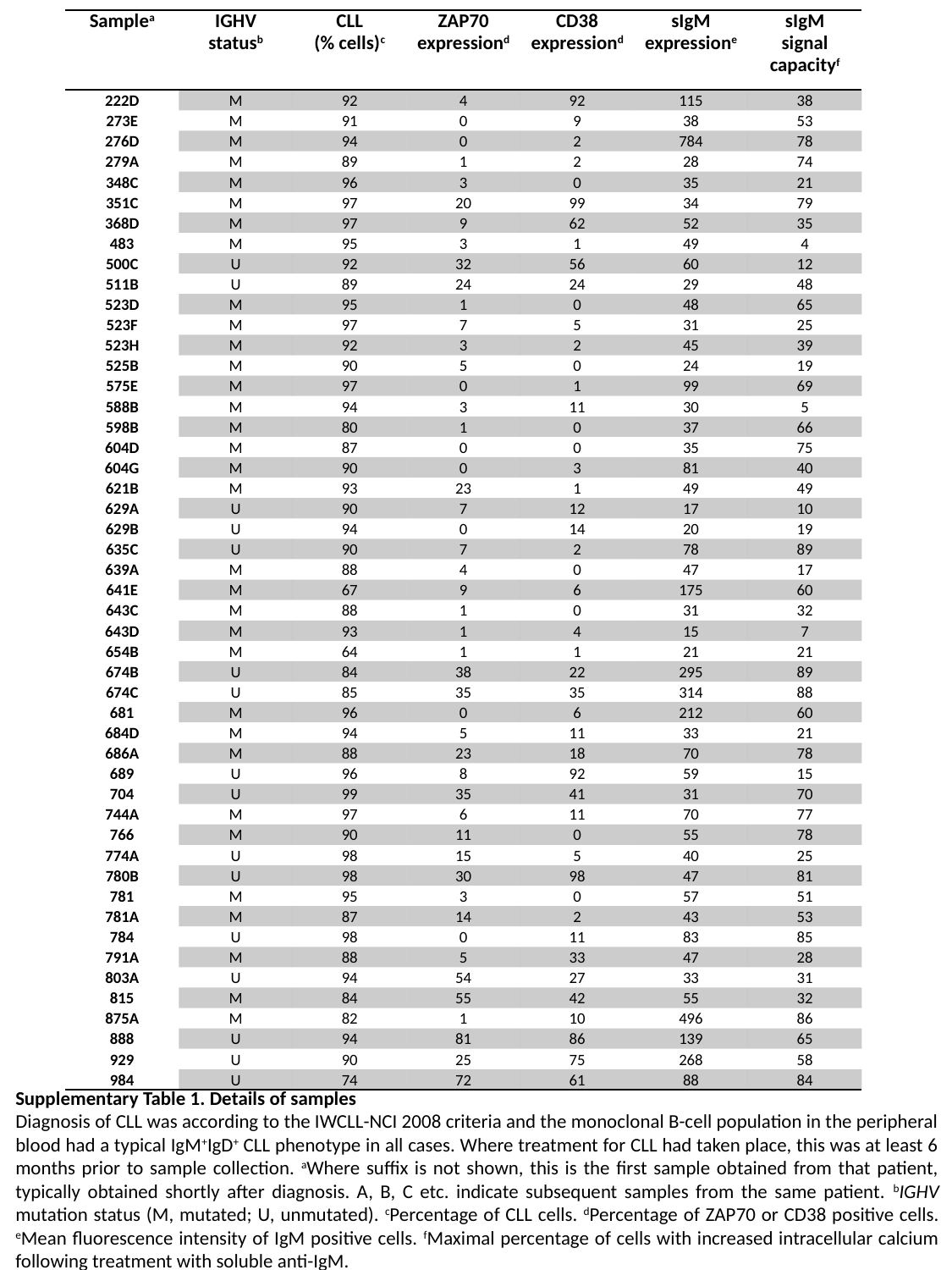

| Samplea | IGHV statusb | CLL (% cells)c | ZAP70 expressiond | CD38 expressiond | sIgM expressione | sIgM signal capacityf |
| --- | --- | --- | --- | --- | --- | --- |
| 222D | M | 92 | 4 | 92 | 115 | 38 |
| 273E | M | 91 | 0 | 9 | 38 | 53 |
| 276D | M | 94 | 0 | 2 | 784 | 78 |
| 279A | M | 89 | 1 | 2 | 28 | 74 |
| 348C | M | 96 | 3 | 0 | 35 | 21 |
| 351C | M | 97 | 20 | 99 | 34 | 79 |
| 368D | M | 97 | 9 | 62 | 52 | 35 |
| 483 | M | 95 | 3 | 1 | 49 | 4 |
| 500C | U | 92 | 32 | 56 | 60 | 12 |
| 511B | U | 89 | 24 | 24 | 29 | 48 |
| 523D | M | 95 | 1 | 0 | 48 | 65 |
| 523F | M | 97 | 7 | 5 | 31 | 25 |
| 523H | M | 92 | 3 | 2 | 45 | 39 |
| 525B | M | 90 | 5 | 0 | 24 | 19 |
| 575E | M | 97 | 0 | 1 | 99 | 69 |
| 588B | M | 94 | 3 | 11 | 30 | 5 |
| 598B | M | 80 | 1 | 0 | 37 | 66 |
| 604D | M | 87 | 0 | 0 | 35 | 75 |
| 604G | M | 90 | 0 | 3 | 81 | 40 |
| 621B | M | 93 | 23 | 1 | 49 | 49 |
| 629A | U | 90 | 7 | 12 | 17 | 10 |
| 629B | U | 94 | 0 | 14 | 20 | 19 |
| 635C | U | 90 | 7 | 2 | 78 | 89 |
| 639A | M | 88 | 4 | 0 | 47 | 17 |
| 641E | M | 67 | 9 | 6 | 175 | 60 |
| 643C | M | 88 | 1 | 0 | 31 | 32 |
| 643D | M | 93 | 1 | 4 | 15 | 7 |
| 654B | M | 64 | 1 | 1 | 21 | 21 |
| 674B | U | 84 | 38 | 22 | 295 | 89 |
| 674C | U | 85 | 35 | 35 | 314 | 88 |
| 681 | M | 96 | 0 | 6 | 212 | 60 |
| 684D | M | 94 | 5 | 11 | 33 | 21 |
| 686A | M | 88 | 23 | 18 | 70 | 78 |
| 689 | U | 96 | 8 | 92 | 59 | 15 |
| 704 | U | 99 | 35 | 41 | 31 | 70 |
| 744A | M | 97 | 6 | 11 | 70 | 77 |
| 766 | M | 90 | 11 | 0 | 55 | 78 |
| 774A | U | 98 | 15 | 5 | 40 | 25 |
| 780B | U | 98 | 30 | 98 | 47 | 81 |
| 781 | M | 95 | 3 | 0 | 57 | 51 |
| 781A | M | 87 | 14 | 2 | 43 | 53 |
| 784 | U | 98 | 0 | 11 | 83 | 85 |
| 791A | M | 88 | 5 | 33 | 47 | 28 |
| 803A | U | 94 | 54 | 27 | 33 | 31 |
| 815 | M | 84 | 55 | 42 | 55 | 32 |
| 875A | M | 82 | 1 | 10 | 496 | 86 |
| 888 | U | 94 | 81 | 86 | 139 | 65 |
| 929 | U | 90 | 25 | 75 | 268 | 58 |
| 984 | U | 74 | 72 | 61 | 88 | 84 |
Supplementary Table 1. Details of samples
Diagnosis of CLL was according to the IWCLL-NCI 2008 criteria and the monoclonal B-cell population in the peripheral blood had a typical IgM+IgD+ CLL phenotype in all cases. Where treatment for CLL had taken place, this was at least 6 months prior to sample collection. aWhere suffix is not shown, this is the first sample obtained from that patient, typically obtained shortly after diagnosis. A, B, C etc. indicate subsequent samples from the same patient. bIGHV mutation status (M, mutated; U, unmutated). cPercentage of CLL cells. dPercentage of ZAP70 or CD38 positive cells. eMean fluorescence intensity of IgM positive cells. fMaximal percentage of cells with increased intracellular calcium following treatment with soluble anti-IgM.

## Slide 3
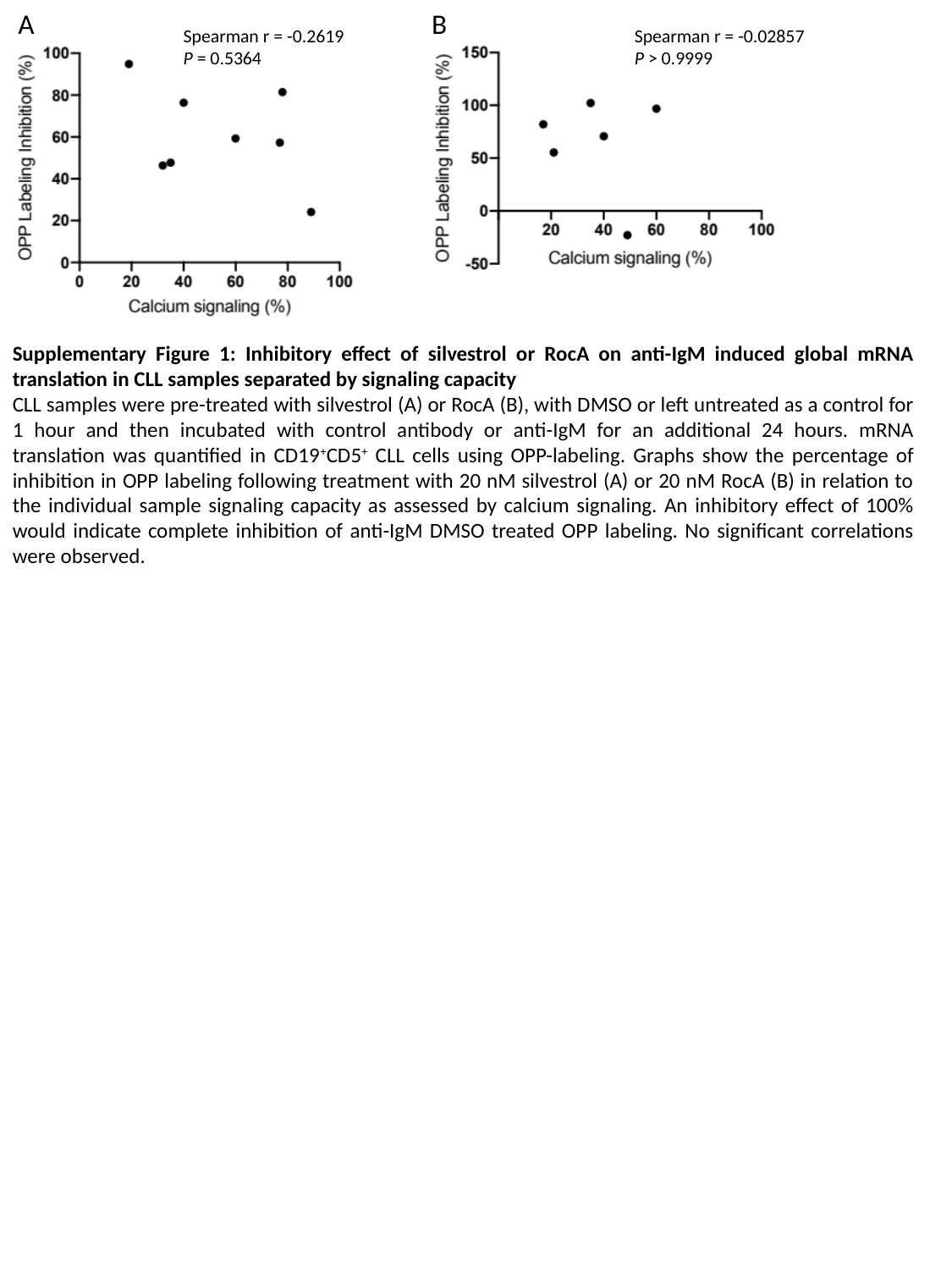

A
B
Spearman r = -0.02857
P > 0.9999
Spearman r = -0.2619
P = 0.5364
Supplementary Figure 1: Inhibitory effect of silvestrol or RocA on anti-IgM induced global mRNA translation in CLL samples separated by signaling capacity
CLL samples were pre-treated with silvestrol (A) or RocA (B), with DMSO or left untreated as a control for 1 hour and then incubated with control antibody or anti-IgM for an additional 24 hours. mRNA translation was quantified in CD19+CD5+ CLL cells using OPP-labeling. Graphs show the percentage of inhibition in OPP labeling following treatment with 20 nM silvestrol (A) or 20 nM RocA (B) in relation to the individual sample signaling capacity as assessed by calcium signaling. An inhibitory effect of 100% would indicate complete inhibition of anti-IgM DMSO treated OPP labeling. No significant correlations were observed.

## Slide 4
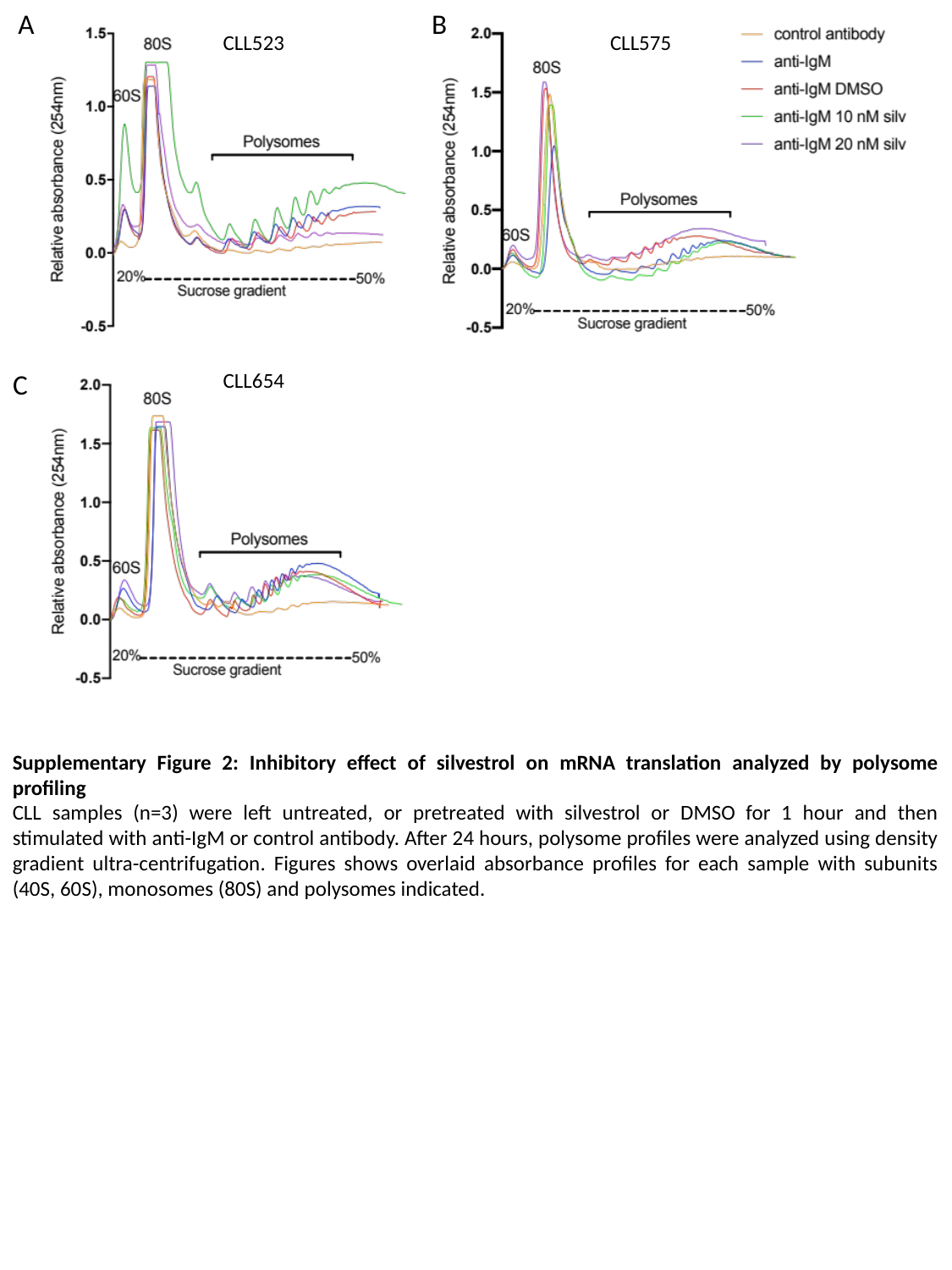

A
B
CLL523
CLL575
C
CLL654
Supplementary Figure 2: Inhibitory effect of silvestrol on mRNA translation analyzed by polysome profiling
CLL samples (n=3) were left untreated, or pretreated with silvestrol or DMSO for 1 hour and then stimulated with anti-IgM or control antibody. After 24 hours, polysome profiles were analyzed using density gradient ultra-centrifugation. Figures shows overlaid absorbance profiles for each sample with subunits (40S, 60S), monosomes (80S) and polysomes indicated.

## Slide 5
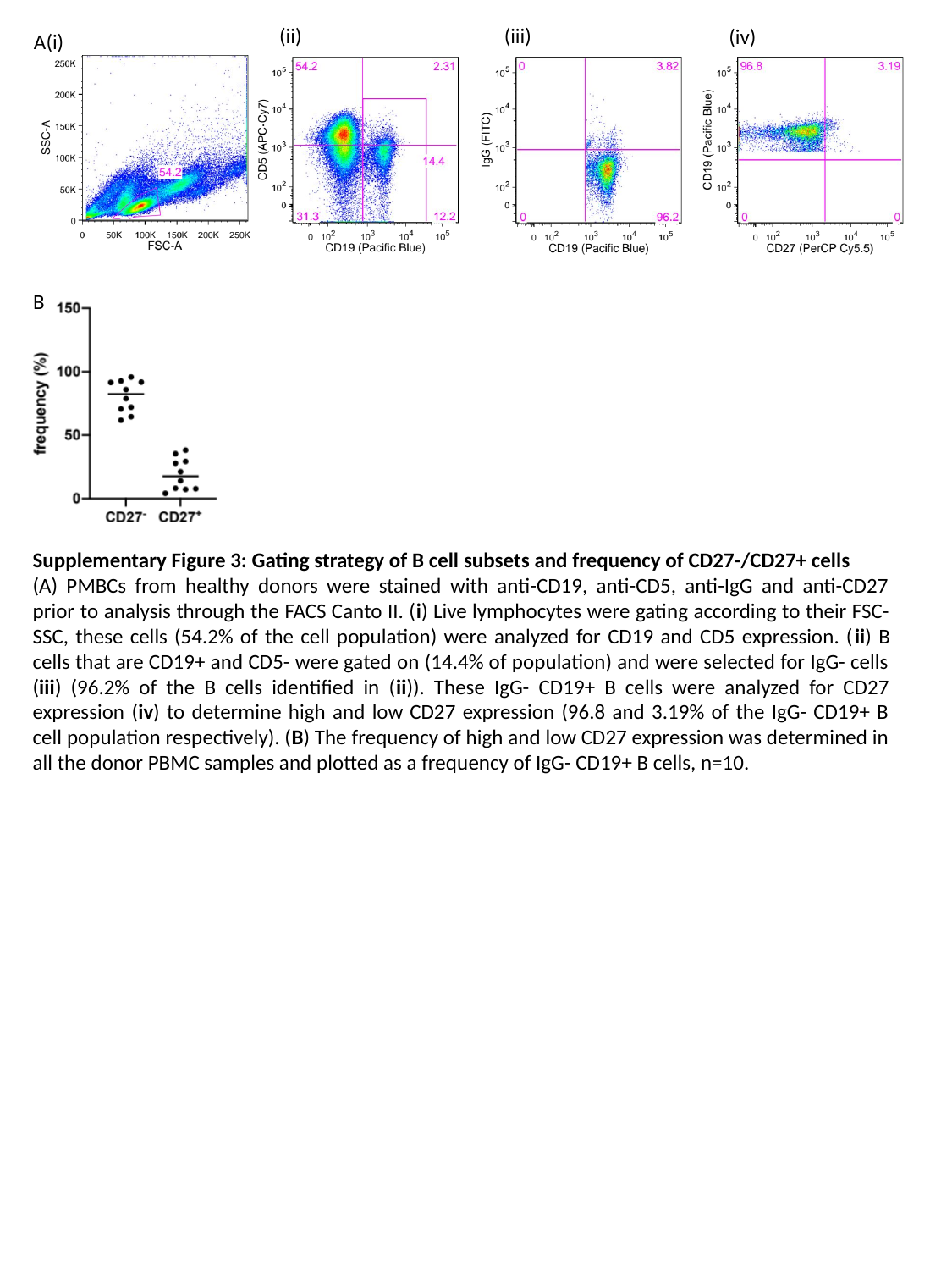

(ii)
(iii)
(iv)
A(i)
B
Supplementary Figure 3: Gating strategy of B cell subsets and frequency of CD27-/CD27+ cells
(A) PMBCs from healthy donors were stained with anti-CD19, anti-CD5, anti-IgG and anti-CD27 prior to analysis through the FACS Canto II. (i) Live lymphocytes were gating according to their FSC-SSC, these cells (54.2% of the cell population) were analyzed for CD19 and CD5 expression. (ii) B cells that are CD19+ and CD5- were gated on (14.4% of population) and were selected for IgG- cells (iii) (96.2% of the B cells identified in (ii)). These IgG- CD19+ B cells were analyzed for CD27 expression (iv) to determine high and low CD27 expression (96.8 and 3.19% of the IgG- CD19+ B cell population respectively). (B) The frequency of high and low CD27 expression was determined in all the donor PBMC samples and plotted as a frequency of IgG- CD19+ B cells, n=10.

## Slide 6
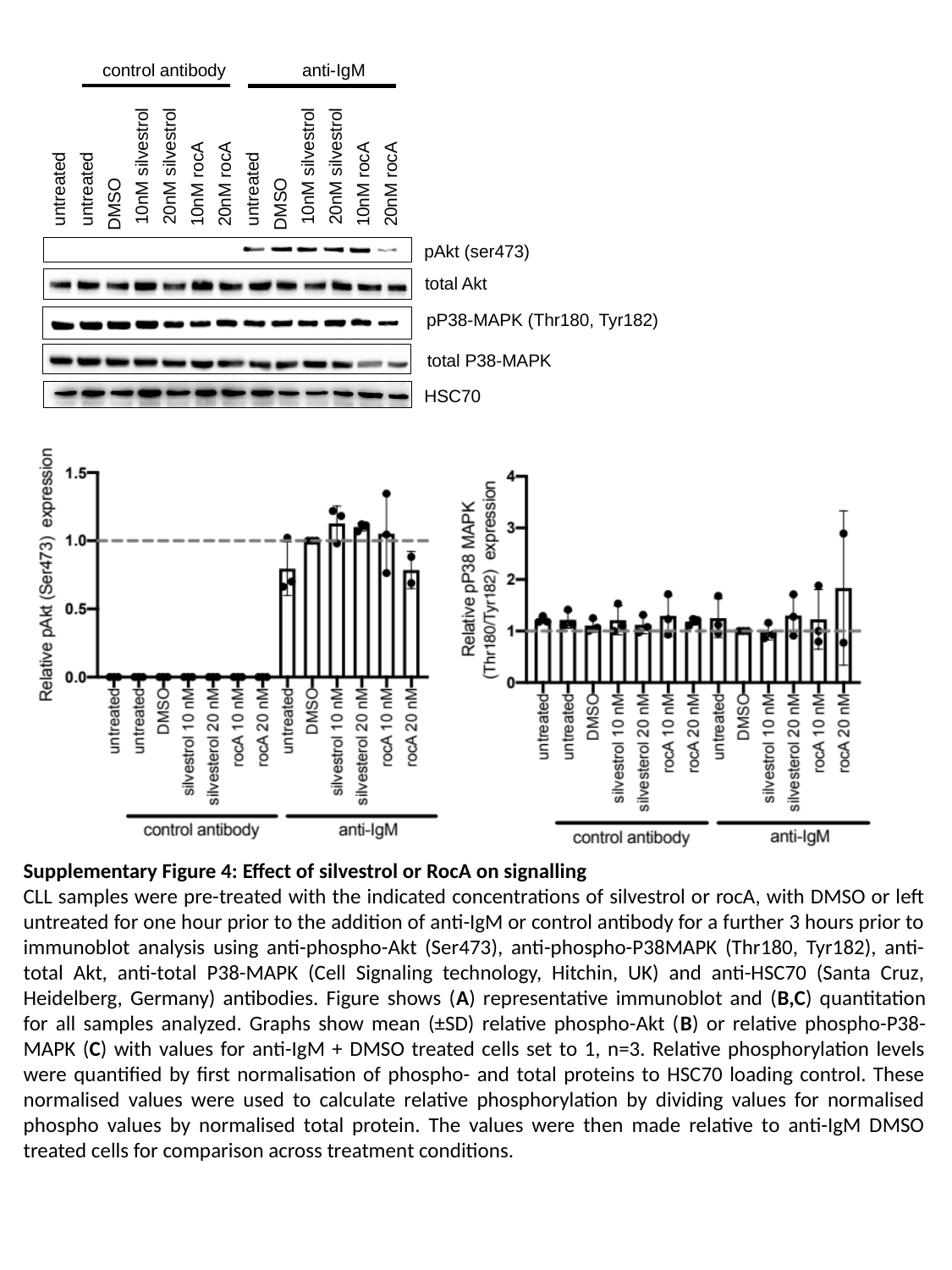

control antibody
anti-IgM
10nM silvestrol
20nM silvestrol
10nM silvestrol
20nM silvestrol
10nM rocA
20nM rocA
10nM rocA
20nM rocA
untreated
untreated
untreated
DMSO
DMSO
pAkt (ser473)
total Akt
pP38-MAPK (Thr180, Tyr182)
total P38-MAPK
HSC70
Supplementary Figure 4: Effect of silvestrol or RocA on signalling
CLL samples were pre-treated with the indicated concentrations of silvestrol or rocA, with DMSO or left untreated for one hour prior to the addition of anti-IgM or control antibody for a further 3 hours prior to immunoblot analysis using anti-phospho-Akt (Ser473), anti-phospho-P38MAPK (Thr180, Tyr182), anti-total Akt, anti-total P38-MAPK (Cell Signaling technology, Hitchin, UK) and anti-HSC70 (Santa Cruz, Heidelberg, Germany) antibodies. Figure shows (A) representative immunoblot and (B,C) quantitation for all samples analyzed. Graphs show mean (±SD) relative phospho-Akt (B) or relative phospho-P38-MAPK (C) with values for anti-IgM + DMSO treated cells set to 1, n=3. Relative phosphorylation levels were quantified by first normalisation of phospho- and total proteins to HSC70 loading control. These normalised values were used to calculate relative phosphorylation by dividing values for normalised phospho values by normalised total protein. The values were then made relative to anti-IgM DMSO treated cells for comparison across treatment conditions.

## Slide 7
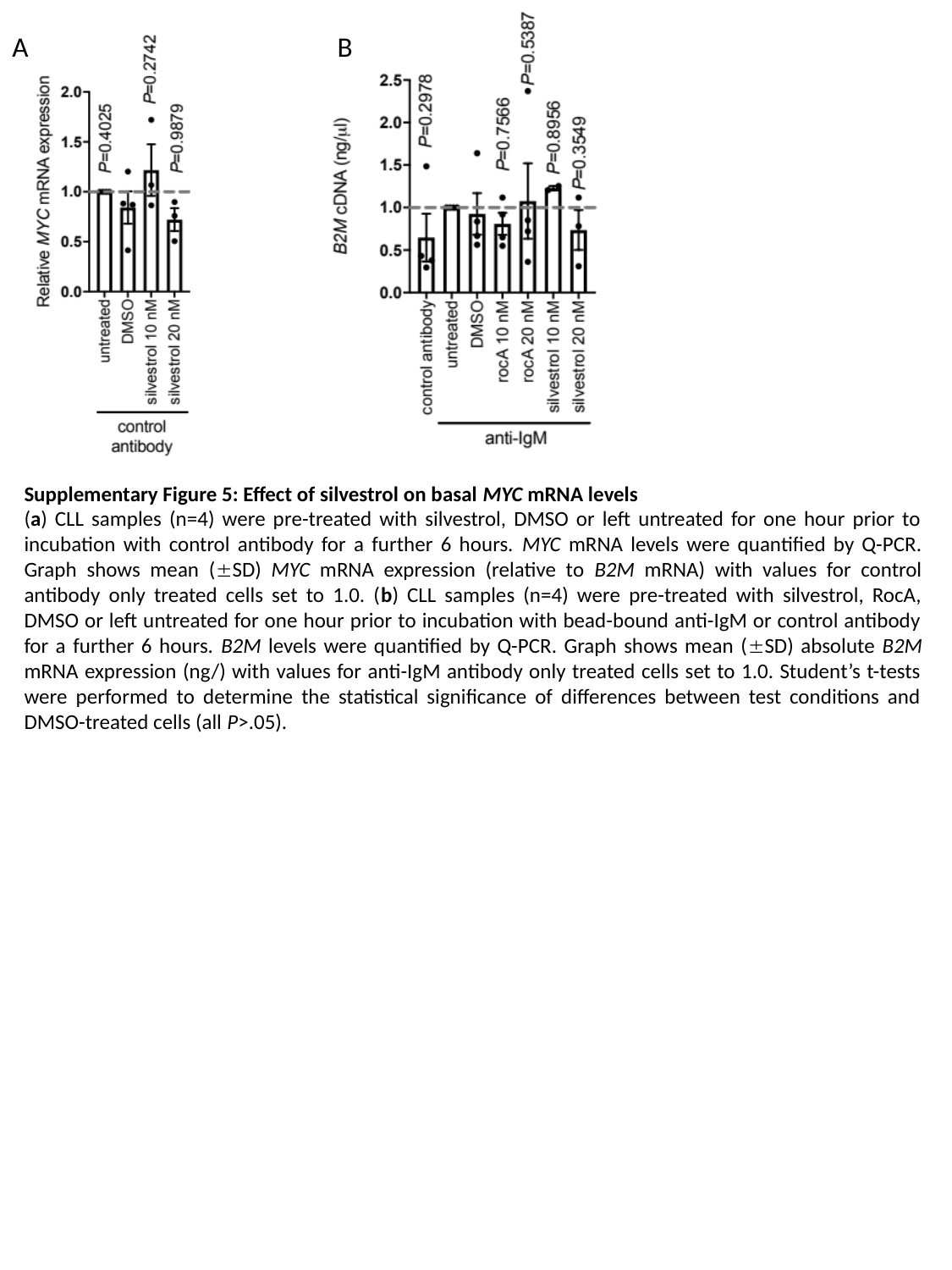

A
B

## Slide 8
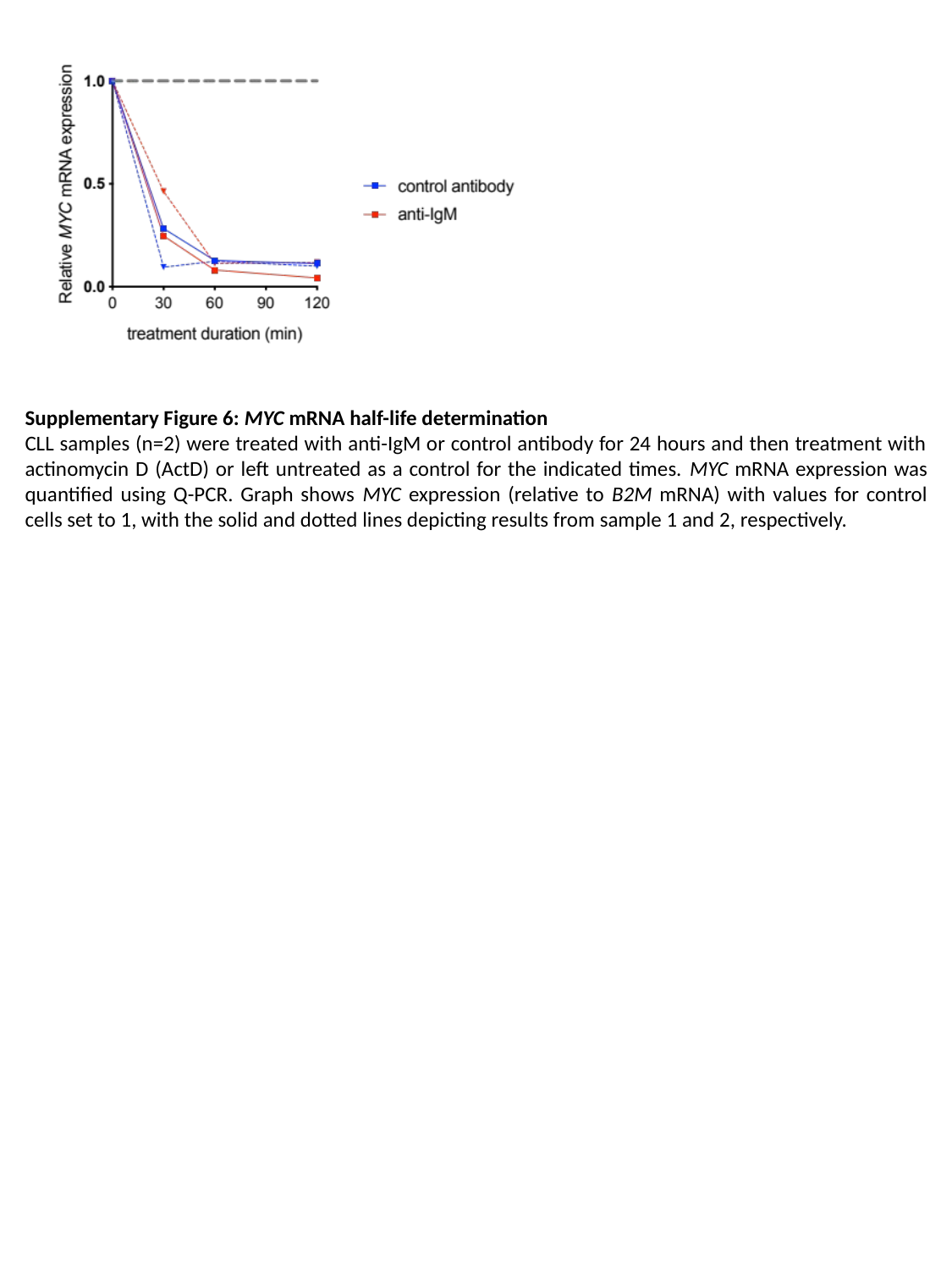

Supplementary Figure 6: MYC mRNA half-life determination
CLL samples (n=2) were treated with anti-IgM or control antibody for 24 hours and then treatment with actinomycin D (ActD) or left untreated as a control for the indicated times. MYC mRNA expression was quantified using Q-PCR. Graph shows MYC expression (relative to B2M mRNA) with values for control cells set to 1, with the solid and dotted lines depicting results from sample 1 and 2, respectively.

## Slide 9
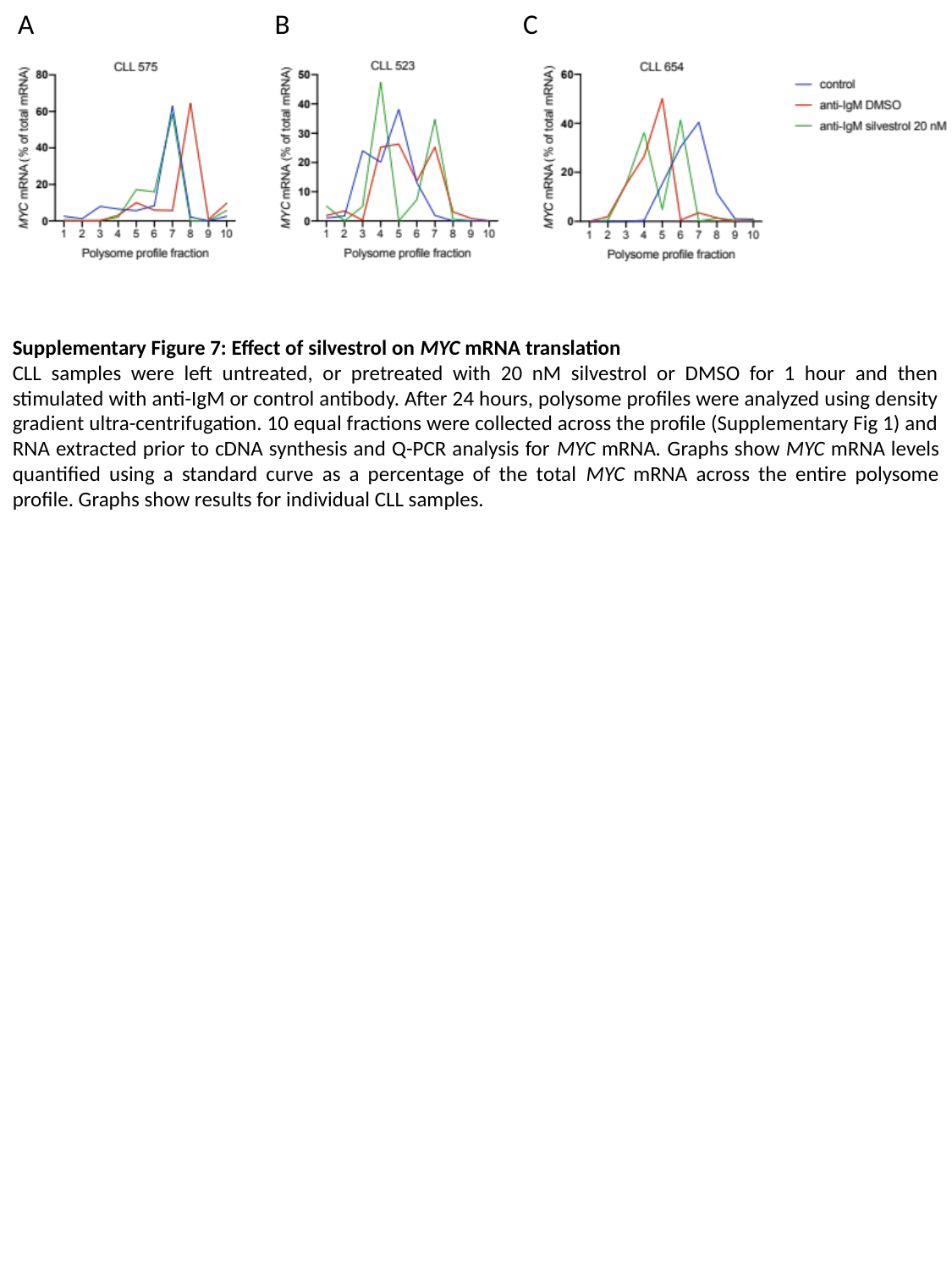

B
C
A
Supplementary Figure 7: Effect of silvestrol on MYC mRNA translation
CLL samples were left untreated, or pretreated with 20 nM silvestrol or DMSO for 1 hour and then stimulated with anti-IgM or control antibody. After 24 hours, polysome profiles were analyzed using density gradient ultra-centrifugation. 10 equal fractions were collected across the profile (Supplementary Fig 1) and RNA extracted prior to cDNA synthesis and Q-PCR analysis for MYC mRNA. Graphs show MYC mRNA levels quantified using a standard curve as a percentage of the total MYC mRNA across the entire polysome profile. Graphs show results for individual CLL samples.
